# Management of Primary Mediastinal B-Cell Lymphoma in Pregnancy

**DOI:** 10.7759/cureus.2215

**Published:** 2018-02-21

**Authors:** Sidra Khalid, Aariez Khalid, Hamed Daw, Praful Maroo

**Affiliations:** 1 Internal Medicine Residency, Fairview Hospital, Cleveland Clinic, USA; 2 Bachelor of Science (biomedical Science), University of Guelph; 3 Department of Hematology and Oncology, Fairview Hospital, Cleveland Clinic, USA; 4 Cardiology, Fairview Hospital, Cleveland Clinic, USA

**Keywords:** pmbcl, pregnancy, rchop, rice, radiation, fetal

## Abstract

Primary mediastinal B-cell lymphoma (PMBCL) is a subtype of non-Hodgkin’s lymphoma, which occurs rarely in pregnancy. We present a case of a pregnant 22-year-old female who presented with syncope and dyspnea. Computed tomography (CT) chest showed an anterior mediastinal mass, and its biopsy showed PMBCL. Since she was in her second trimester, we decided to treat her with rituximab-cyclophosphamide, doxorubicin, vincristine, prednisone (R-CHOP). Our case emphasizes the safety of chemotherapy in the second and third trimesters, with good maternal and fetal outcomes.

## Introduction

Primary mediastinal B-cell lymphoma (PMBCL) occurs in about 2.5% of cases of non-Hodgkin’s lymphoma. It is predominant in the third and fourth decade of life, commonly occurring in the reproductive age group in females [1]. It could occur during pregnancy, presenting with local compression symptoms. Management involves chemotherapy. During pregnancy, in the second and third trimesters, rituximab-cyclophosphamide, doxorubicin, vincristine, prednisone (R-CHOP) could be administered with favorable maternal and fetal outcomes. Our case focuses on the multidisciplinary approach for managing PMBCL in a pregnant female.

## Case presentation

**Figure 1:**
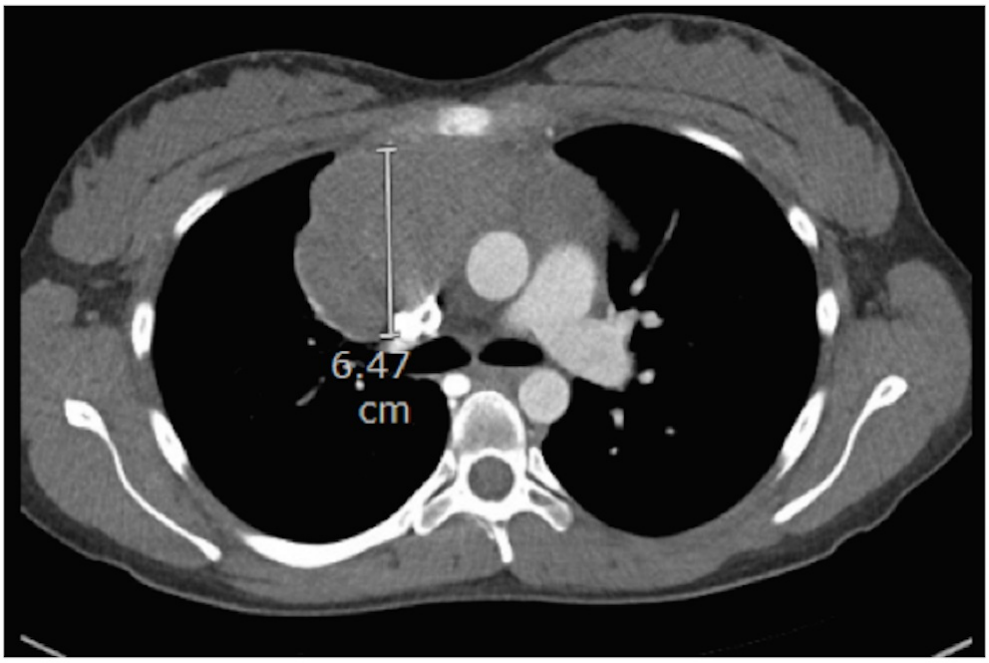
CT chest showing an anterior mediastinal mass measuring 6.47 cm

A 22-year-old female, gravida 3 para 2 at 22 weeks' gestation, was referred to a cardiology clinic for syncope, cough, and dyspnea on exertion. Her vital signs were BP: 116/50 mmHg, heart rate (HR): 101/min, respiratory rate (RR): 21/min, afebrile, and peripheral capillary oxygen saturation (SpO2): 96%. Her physical examination was significant for marked venous engorgement in the neck, a small palpable lymph node in the right anterior cervical chain, with an abdominal examination of a gravid uterus. A chest x-ray showed an abnormal left cardiac contour with a prominence of both hila and cardiomegaly. She was admitted to intensive care. A computed tomography (CT) scan of the chest showed a large anterior mediastinal mass, 15.9 x 8.0 x 12.1 cm, with mass effect upon the superior vena cava, which was narrowed but patent; with axillary lymph nodes bilaterally, 1.1 cm in size (Figure 1). There was no adenopathy in the magnetic resonance imaging (MRI) abdomen. A CT guided biopsy was performed of the mediastinal mass and the results were pending. The differential diagnosis included lymphoma and she was started on prednisone 1 mg/kg and then discharged on prednisone 60 mg daily for symptomatic relief for shortness of breath.

**Figure 2:**
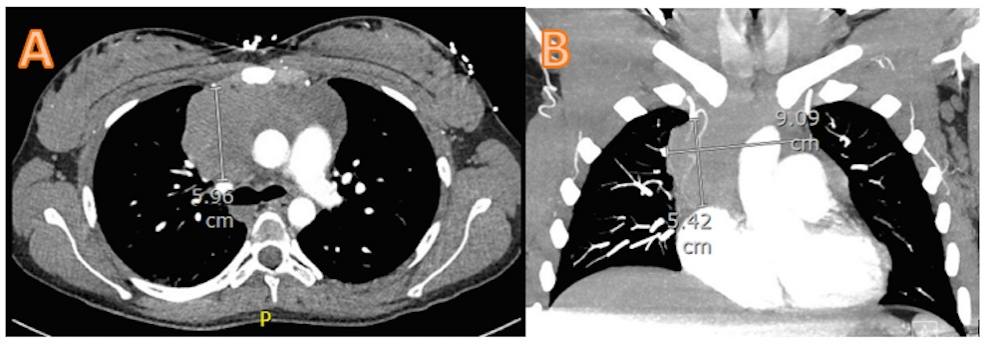
CT chest showing the decreased size of the anterior mediastinal mass: 5.96 cm in sagittal section (A) and 5.42 x 9.09 cm in coronal section (B)

After four days, she returned to the emergency department with worsening dyspnea, cough, and wheezing. She was admitted to the intensive care unit since the previous CT scan of the chest showed that the mass was compressing the right hilar and subcarinal regions. During the admission, the results of the biopsy revealed PMBCL. She was given intravenous methylprednisolone, started on allopurinol, and a mediport was inserted through the common femoral vein into the inferior vena cava. She was started on treatment for stage IIA PMBCL with cycle # 1 R-CHOP. At 25 weeks' gestation, she received cycle #2 and her symptoms of dyspnea and neck engorgement largely resolved. At 28 weeks, cycle #3 was given. At 31 weeks, she presented to the emergency department with worsening dyspnea and orthopnea. The electrocardiogram showed sinus tachycardia of 109/min. CT chest indicated a subsegmental filling defect in the right lower lobe, which was suggestive of a pulmonary embolus, and a decreased size of the mediastinal mass (Figure 2 A, B); ultrasound of lower extremities revealed no deep venous thrombosis. Previously, she was on enoxaparin 40 mg daily, but after the diagnosis of pulmonary embolism, she was started on enoxaparin 60 mg q 12 hrs. She received two more cycles of RCHOP, with cycle #5 at 35 weeks. She went into labor at 37 weeks, was on heparin for pulmonary embolism, and underwent a spontaneous vaginal delivery of a viable male infant. The Apgar score was 8. She was discharged home and was advised to obtain a positron emission tomography (PET) scan for the restaging of the PMBCL, a CT chest and abdomen, and to switch to warfarin for pulmonary embolism. On the follow-up appointment, after completing five cycles of R-CHOP, her CT chest showed an increase in the size of the anterior mediastinal mass to 6.5 x 8.9 x 10.1 cm. The biopsy of the mass was consistent with PMBCL, with the cells expressing CD20, PAX5, and CD30 and having a Ki67 index of 90%. She had refractory PMBCL and was given rituxan, ifosfamide, carboplatin, etoposide (RICE) for three weeks followed by autologous stem cell transplant. Afterwards, on CT chest, the mass, was 3.8 x 2.6 cm in size and she underwent radiation to the neck, mediastinum and both axillae. Her anterior mediastinal mass was stable at a size of 3.7 x 1.5 cm on CT chest with a stable positron emission tomography scan showing non-fludeoxyglucose avid mediastinal soft tissue density. The future plan is to continue observation. 

## Discussion

PMBCL is a subtype of diffuse large B cell lymphoma that arises from thymic B-cells. It usually manifests itself during pregnancy at a median 24 weeks of gestation [1]. As it is a bulky mediastinal tumor, it presents with local compressive symptoms, such as dyspnea, cough, dysphagia, and superior vena cava obstruction [2]. In about 50% of the cases, there could be pleural or pericardial effusions. On pathology, there are thymic B-cells, which medium-large cells with abundant pale cytoplasm and delicate interstitial fibrosis. On immunochemistry, B-cell antigens are present; these are CD19, CD20, CD22, CD79a, and CD30 [1]. 

The management of PMBCL involves chemotherapy and radiation. Cyclophosphamide, doxorubicin, vincristine, prednisone (CHOP) achieves a cure rate of 50%-60%. When rituximab is added to the CHOP regimen, cure rates exceeding 82% are achieved [1]. In a study by Mangasarova et al., eight pregnant females with PMBCL underwent induction chemotherapy in their second and third trimesters. Etoposide, doxorubicin, cyclophosphamide, vincristine, prednisone, bleomycin (VACOPB) in four and rituximab and dose-adjusted etoposide, vincristine, doxorubicin, cyclophosphamide, prednisone (R+DAEPOCH) in three patients. Among these, 6/8 achieved partial remission, 1/8 had progression, and 1/8 had complete remission. High-dose chemotherapy was given to 7/8 women after delivery, followed by radiation. The newborns of females who were given rituximab developed pneumonia. Therefore, this study showed that chemotherapy could be given to pregnant females in the second and third trimesters [3].

Another regimen, methotrexate, cytarabine, cyclophosphamide, vincristine, prednisone, bleomycin (MACOP-B) along with field radiation has achieved complete remission rates of 86%. The EPOCH regimen, with and without rituximab, achieved overall survival of 100% and event-free survival of 94%. Due to the lack of evidence of the MACOP-B and EPOCH regimens in pregnancy, they are not used at this time [1].

In addition, there is an increased risk of thromboembolism in patients with PMBCL. Lekovic et al. conducted a retrospective study of 42 patients with PMBCL and identified that 15/42 had thrombosis. 14 had deep venous thrombosis and one had a pulmonary embolism. They noted that patients with thrombosis had bulky mediastinal masses, and it adversely affected their survival. They concluded that prophylactic anticoagulation should be offered to PMBCL patients with chemotherapy to prevent thrombotic events [4].

## Conclusions

PMBCL is an uncommon hematologic malignancy that may be present during pregnancy. A multidisciplinary approach, including oncologists, radiologists, obstetricians, fetal medicine specialists, social services, and, in this case, cardiologists, is critical to optimize staging and treatment plans. In our case, we highlighted that pregnant patients may safely receive chemotherapy for the treatment of PMBCL during the two to three trimesters, with favorable maternal and fetal outcomes.
